# Realistic three-dimensional avian vocal tract model demonstrates how shape affects sound filtering (*Passer domesticus*)

**DOI:** 10.1098/rsif.2022.0728

**Published:** 2023-01-25

**Authors:** Alireza Kazemi, Mariam Kesba, Pauline Provini

**Affiliations:** ^1^ Inserm, System Engineering and Evolution Dynamics, Université Paris Cité, 75004 Paris, France; ^2^ Learning Planet Institute, 75004 Paris, France; ^3^ Département Adaptations du Vivant, UMR MECADEV 7179 CNRS/Muséum National d'Histoire Naturelle, Paris, France

**Keywords:** frequency modulation, vocal tract filter, bioacoustics, oropharyngeal and oesophageal cavity, Passeriformes

## Abstract

Despite the complex geometry of songbird's vocal system, it was typically modelled as a tube or with simple mathematical parameters to investigate sound filtering. Here, we developed an adjustable computational acoustic model of a sparrow's upper vocal tract (*Passer domesticus*), derived from micro-CT scans. We discovered that a 20% tracheal shortening or a 20° beak gape increase caused the vocal tract harmonic resonance to shift toward higher pitch (11.7% or 8.8%, respectively), predominantly in the mid-range frequencies (3–6 kHz). The oropharyngeal-oesophageal cavity (OEC), known for its role in sound filtering, was modelled as an adjustable three-dimensional cylinder. For a constant OEC volume, an elongated cylinder induced a higher frequency shift than a wide cylinder (70% versus 37%). We found that the OEC volume adjustments can modify the OEC first harmonic resonance at low frequencies (1.5–3 kHz) and the OEC third harmonic resonance at higher frequencies (6–8 kHz). This work demonstrates the need to consider the realistic geometry of the vocal system to accurately quantify its effect on sound filtering and show that sparrows can tune the entire range of produced sound frequencies to their vocal system resonances, by controlling the vocal tract shape, especially through complex OEC volume adjustments.

## 1. Introduction

In both birds and humans, the sound produced by the vocal organs can be modified by complex filtering effects throughout the vocal tract. In humans, sounds generated by the vocal cords, in the larynx, are modulated by the shape and volume variations of several structures of the upper vocal tract: the trachea, buccal cavity, tongue, lips and nasal cavity [1]. In birds, however, the larynx is not directly associated with sound production [2]. The syrinx, a vibrating organ specific to birds [3–11], produces a primary sound, subsequently filtered by the rest of the vocal tract. Because the syrinx is located near the base of the trachea, several structures, potentially involved in sound filtering [12], need to be crossed before the final sound exits: the trachea, the larynx, through its opening (i.e. the glottis), the beak, with a possible effect of tongue's position.

As described in zebra finches (*Taeniopygia guttata*) and white-throated sparrow (*Zonotrichia albicollis*) [13,14], tracheal length can change during sound production in Passeriformes. However, based on previous numerical models [15], the influence on sound filtering is supposed to be so low that it is usually considered as negligible [13]. Thus, according to past investigations, the tracheal resonance resembles that of a simple tube [14].

On the contrary, numerical models and experiments demonstrated that beak movements can significantly affect acoustic resonance (e.g. [13,15–20]). White-throated sparrows (*Zonotrichia albicollis*) and swamp sparrows (*Melospiza georgiana*) both displayed a positive correlation between acoustic frequencies and beak opening during the first two notes of their songs [20]. In the adult male northern cardinals (*Cardinalis cardinalis*) high harmonic resonance frequencies are associated with a wide beak gape [19]. The strongest tuning effect of the beak occurs when it is nearly closed [15,21]. In addition, experimental data showed that longer beaks attenuate high-frequency sounds produced by the syrinx [15,22]. However, these results are based on correlations between beak opening and recorded frequencies on a relatively small number of bird species, therefore the exact influence of beak movements on sound filtering remain uncertain.

The tongue also has the potential to modify harmonic resonance frequencies. In the northern cardinal (*Cardinalis cardinalis*), the tip of the tongue can be lifted until touching the palate, narrowing the airflow from the oral cavity into the surrounding environment. Below 2 kHz, tongue rise decreases the harmonic resonance frequency of the vocal tract [23]. Similarly, in the monk parakeet (*Myiopsitta monachus*), tongue position is positively correlated with amplitude variations [9].

Because most birds lack a soft palate, there is no clear demarcation between the roof of the mouth and the pharynx, forming the oropharynx cavity [24]. The oropharynx cavity opens to the larynx, leading to the trachea, through the glottis, and more dorsally to the oesophagus [24]. X-ray cinematography of the vocal tract of Passeriformes showed that during song, the oropharyngeal and oesophageal cavity (OEC) can suddenly and consistently increase volume [10,14,15,19,25,26], through the movement of the hyoid and larynx. Cineradiography of the northern cardinal and sparrow showed an inverse relationship between the OEC volume and the harmonic resonance frequency: at low frequencies, the OEC tends to remain at a maximum volume, but at higher frequencies, the OEC collapses to a minimum volume [14,19]. Using a first-order model in which OEC is assumed to be a cylinder, with the glottis close to the midpoint [15], the relative resonance was calculated as a function of frequency [15]. This model proved that the OEC can add harmonic resonance to the vocal tract, acting as an acoustic bandpass filter. Although this model adds valuable knowledge to our understanding of bird vocalization, the total volume of the OEC was considered as a single parameter, thus the potential effect of its complex shape was ignored, and the model was not accurate for frequencies occurring when the wavelength was smaller than approximately twice the OEC length [15].

Investigating the influence of the intrinsic shape and deformations of each part of the upper vocal system is a challenge in experimental conditions, therefore modelling approaches are necessary to address those limits. The most advanced model, largely used to date, is based on an electrical network analogue model, and by construction, ignores the effect of the organs' real shape [14,15,19,21]. Here, we focused on the house sparrow (*Passer domesticus*), a common model species in acoustic analyses [14,18,20,27–29]. Its song consists of a series of constant frequency whistle notes, which facilitates simulations. We took advantage of the recent advances in imaging, especially in contrast-enhanced micro-computed tomography (µCT) scans [30] to build a realistic anatomical three-dimensional model of the house sparrow's vocal system, including the hard and soft tissues of the trachea, larynx, tongue and beak. Using this initial model, we computed the total sound pressure level on an arbitrary sound produced by a virtual syrinx. We built four types of models by modifying the shape of the four vocal structures of interest, all other factors remaining equal, to investigate the influence of each vocal structures on sound modulation.

We also integrated the OEC to our realistic computational acoustic model. Considering that the real OEC shape is dynamically changing during vocalization and is thus not directly available on CT scans, we mimicked the shape of extreme OEC volumes during sound production [14,15], using a cylindrical model. We constructed several models with different shapes but similar volumes, comparable to those used in prior works [14,15]. This allowed us to test the influence of the cylinder's length and diameter, thus the influence of the real cylinder shape, for a given volume.

We mimic the many conformations the upper vocal tract can take during vocalization by changing the geometry of the various upper vocal tract components. Since most aspects of human and songbird vocal behaviour have to be learned, including vocal tract movements, assessing the influence of each component on sound filtering is an important prerequisite to understand underlying neural control of those movements and have a better understanding of bird complex communication, one of the amazing characteristics we share with them.

## 2. Results

### 2.1. Computed tomography scan

**Figure 1.**
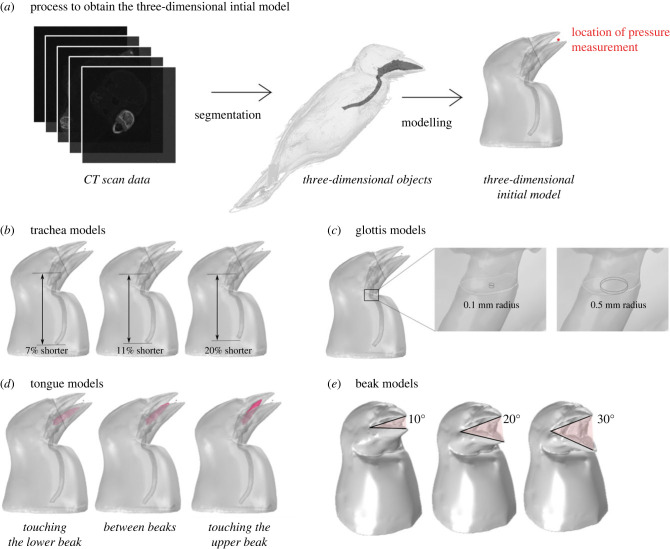
Sketch showing the steps to obtain the models used in the study (*a*) and the four types of realistic three-dimensional shape models used in the analysis: the trachea models (*b*), the glottis models (*c*), the tongue models (*d*) and the beak models (*e*).

We built an initial model derived from µCT scan data. We modified its geometry to test the influence of the trachea length, glottis diameter, beak gape and tongue position, on sound filtering (figure 1).

### 2.2. Initial model

**Figure 2.**
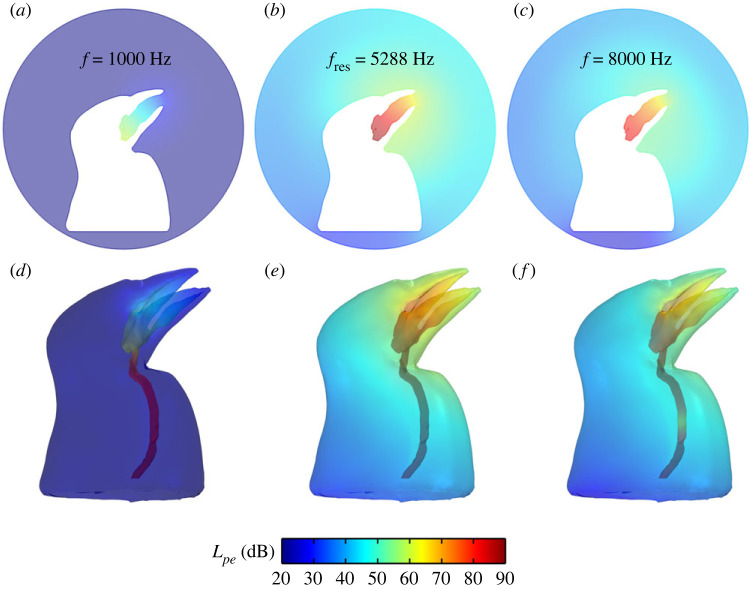
View of the total external sound pressure level (Lp) in dB in the atmosphere for the sparrow initial model with 10° beak gape, at the following tested frequencies: (*a*) 1 kHz, (*b*) 5.28 kHz (harmonic resonance frequency), (*c*) 8 kHz, and the internal sound pressure level for the sparrow vocal system in (*d*) 1 kHz, (*e*) 5.28 kHz, (*f*) 8 kHz.

The initial model has a 30.58 mm trachea and a 10° beak gape. The total sound pressure level of the sparrow's initial model is computed within the typical range of frequency of a house sparrow song (1 to 8 kHz) [31]. We simulated the sound pressure level (dB) in the atmosphere surrounding the bird's body (figure 2*a–c*) and inside the bird's vocal system and body (figure 2*d–f*). In this model, we only observed one harmonic resonance, which is related to the length of the trachea. The harmonic resonance frequency is at 5.28 kHz with a 61 dB amplitude (figure 2*b,e*). At 1 kHz, the lowest sound pressure simulated, we measured an amplitude of 30 dB (figure 2*a,d*), which is 31 dB lower than the pressure at which the resonance frequency was observed. At 8 kHz, the highest sound pressure tested, the amplitude was 5 dB lower than the harmonic resonance frequency (figure 2*c,f*). For all tested frequencies, the loudest total sound pressure level around the boundary layer was also related to the 5.28 kHz harmonic resonance frequency.

### 2.3. Influence of tracheal length

**Figure 3.**
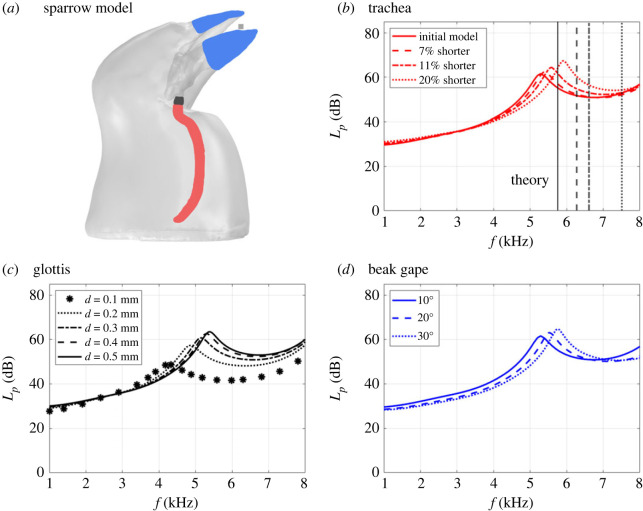
(*a*) Sparrow model with the trachea (red), glottis (black) and beak (blue). Total sound pressure level in dB (Lp) versus frequencies (*f*) from 1 to 8 kHz, for (*a*) trachea (the vertical lines indicate the theoretical harmonic resonance for a perfect cylindrical tube of the different lengths), (*b*) glottis and (*c*) beak at different gape angles.

To test the effects of the tracheal length, we decrease the initial tracheal length by 7%, 11% and 20%, keeping a consistent diameter of approximately 1 mm. The simulation (figure 3*b*) shows a shift of harmonic resonance frequency from 5.28 kHz to 5.40, 5.58 and 5.91 kHz and from 61 dB to 62, 64 and 67 dB in amplitude for the 7%, 11% and 20% shorter models, respectively. The vertical line in figure 3*b* indicates the theoretical harmonic resonance for a perfect cylindrical tube with the same length.

Ignoring the end correction effect, the theory predicts an inverse linear relationship between a perfect tube length and the resonance (equation (4.1)). We obtained an increase of the harmonic resonance frequency when decreasing the tube length, but because of the effect of three-dimensional morphology and other organs, the relationship is not purely linear.

### 2.4. Influence of glottis radius

An increase of the glottis radius leads to an increase in both harmonic resonance frequency and amplitude (figure 3*c*). The glottis model with a 0.5 mm radius shows an increase of 1.11 kHz (26%) for the frequency and 14 dB (30%) for the amplitude, compared with the 0.1 mm radius.

### 2.5. Influence of tongue position

We found no changes in the total sound pressure level, when tongue position was modified (close to the lower beak, middle of the beaks or close to the upper beak). The harmonic resonance frequency computed is 5.28 kHz.

### 2.6. Influence of beak gape

The maximum total sound pressure level of 5.28 kHz (figure 3*d*) occurs for the smallest beak gape (10°). This corresponds to the harmonic resonance frequency of the vocal tract. With increasing the beak gape, the harmonic resonance frequency shifts forward, until a maximum of 5.53 kHz and 5.75 kHz for 20° and 30°, respectively. Our results follow a linear variation.

### 2.7. Influence of oropharyngeal-oesophageal cavity, controlled by length

**Figure 4.**
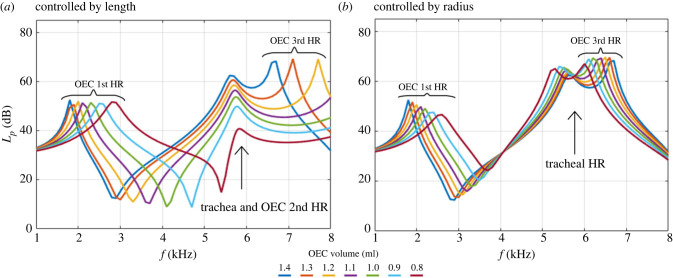
Total sound pressure level in the scale of dB versus frequency from 1 to 8 kHz. Sparrow model with seven different OEC volumes. (*a*) Volume is controlled by the length of the cylinder. (*b*) Volume is controlled by the radius of the cylinder. The colour code in (*a*) and (*b*) represents the same volumes of the OEC, from 0.8 ml to 1.4 ml. The geometry is identical for the volume corresponding to 1.4 ml, with a cylinder length of 20 mm and a radius of 4 mm.

We simulated OEC with seven different internal volumes by changing the length of the cylinder (figure 4*a*). The OEC can add one or two harmonic resonances to the vocal system (depending on the volume) in simulated frequency ranges. The lowest first harmonic resonance of OEC is related to the model with the largest OEC volume (longest OEC). The first harmonic resonance tends to shift toward higher frequencies when the volume of the OEC decreases, and in our models ranged from 1.7 to 2.9 kHz, from the highest (1.4 ml) to the lowest (0.8 ml) OEC volume tested. The first harmonic resonance of the OEC has an identical amplitude for each OEC model of 50 dB.

All models show a common peak in frequency at approximately 5.5 kHz (figure 3), which is associated with the trachea harmonic resonance frequency (indicated with an arrow in figure 4). There is a small acoustic interaction between the OEC volume and the trachea harmonic resonance frequency. A smaller OEC tends to decrease the amplitude of the tracheal resonance. The amplitude of the tracheal harmonic resonance with 0.8 ml OEC is 10 dB lower and the OEC with 1.4 ml is 12 dB higher than the initial model. The third harmonic resonance occurs at a higher range, above 6.6 kHz, and shows an amplitude of 70 dB.

### 2.8. Influence of oropharyngeal-oesophageal cavity, controlled by radius

We simulated OEC with seven different volumes by changing the radius of the cylinder—rather than the length—to investigate the sensitivity of the cylindrical volume and test the influence of the three-dimensional shape on sound modulation (figure 4*b*). When the OEC volume is controlled by radii, it adds a harmonic resonance to the vocal system. The lowest first harmonic resonance of OEC occurs in the model with the largest OEC volume (largest radius). With decreasing the radius (decreasing volume) the first harmonic resonance tends to shift toward higher frequencies, but in different ranges compared with the same volume controlled by length. The first harmonic resonance varies from 1.8 to 2.6 kHz, from the biggest to the smallest OEC radius (from 1.4 to 0.8 ml) with an identical amplitude for each OEC model of approximately 50 dB. All models controlled by radius show a peak between 5.3 and 5.6 kHz (figure 4*b*), which is associated with the harmonic resonance frequency of the trachea (arrow in figure 4*b*). Third OEC harmonic resonance happened in narrower ranges (figure 4*b*) compared with what we observed for the OEC volume controlled by length (figure 4*a*).

## 3. Discussion

According to our computational simulations, the vocal system has the potential to tune the primary produced sound throughout the whole frequency range a house sparrow typically sings (1–8 kHz) [31]. By independently manipulating the geometries of the trachea, glottis and beak gape, using their realistic anatomy, we found that these structures can impact the harmonic content of the mid-frequency range (3–6 kHz). We confirmed that changes in the OEC volume can add at least one harmonic resonance to the vocal system frequencies (below 2 kHz or above 6 kHz) and that the OEC has a dominant influence on sound filtering, compared with the trachea, glottis, tongue and beak. Adding an adjustable OEC revealed that the shape of the OEC (relative length or radius when modelled as a cylinder) affects the modified frequency range. This finding assesses the importance of considering the realistic shape of the vocal system structures to fully quantify their potential in sound filtering.

### 3.1. Trachea

We found that a 7% tracheal shortening resulted in a 2.1% forward shift in harmonic resonance frequency and a 1.6% increase in amplitude (figure 3*b*). The maximum trachea shortening test (20% shortening) shifted the harmonic resonance frequency to 11.7% and increased the amplitude to 9.8%. If we assume a 20% of tracheal elongation, the total tracheal harmonic resonance can vary between 4.6 and 5.9 kHz, corresponding to the middle of the sparrow's vocal frequency range (1–8 kHz) [20,31]. Previous studies found that the trachea elongated by 3% during vocalizations in zebra finches [13] and 11% longitudinal elasticity in the white house sparrow [14], which would result in a relatively low shift of frequency and amplitude. However, the difficulty to accurately measure tracheal length variation prevented us from knowing what the maximum tracheal elongation in a singing bird is. Personal observations on dead birds to quantify the range of motions the vocal tract is following when an operator moves the head/neck structures in different directions, showed a passive trachea elongation of more than 40%.

**Figure 5.**
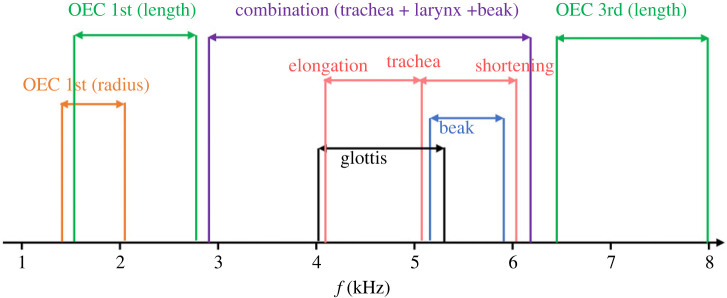
Results from our study suggest that the frequency range (1–8 kHz) of the sparrow's song is produced by multiple parts of the vocal tract anatomy. The harmonic resonance frequency ranges of the OEC first and third harmonic resonance that volume controlled by the length (green), volume controlled by radius (orange), tracheal elongation and shortening model (red), glottis (black), the beak (blue) and combination model (purple).

Theoretical acoustic equations predict an inverse linear relationship between length and harmonic resonance frequency, in a perfect cylinder. In the upper vocal tract model, the vocal system is modelled based on the precise three-dimensional shape derived from µCT scan images. Curves, slight variations in tracheal diameter, ending in the oral cavity, cause a nonlinear correlation between trachea length and harmonic resonance, refining previous modelling of the trachea [14]. Our results show the tracheal harmonic resonance can be tuned with the produced sound in a frequency range between 4 and 6 kHz (figure 5).

### 3.2. Beak

Our simulation results agree with previous experiments [16,20,32]. We found that beak gape slightly affects upper vocal tract resonance. With increasing beak gape from 10° to 30°, the frequency harmonic resonance spans from 5.28 to 5.75 kHz, corresponding to a 9% shift forward. Opening the beak gape by 1° causes a 0.44% frequency shift and a 0.3% amplitude increase. In our model, the lower beak position was modified to increase beak gape; we know that beak kinematics during birdsong is more complex [33]. A detailed kinematic analysis of the different elements of the beak, captured during vocalization would help refine our model.

### 3.3. Glottis

Despite the reduced number of studies of the larynx, we know that the vocal tract diameter decreases when laryngeal muscles contract [34]. To mimic this effect, we modelled the larynx opening, the glottis, at different radii (from 0.1 to 0.5 mm) and measured the impact on frequency and amplitude modulation. Our data revealed that the 0.1 mm wide glottis model showed a harmonic resonance frequency of 4.26 kHz and 59 dB in amplitude (figure 3*c*). The harmonic resonance frequency increased by 26% in the five times wider glottis model, while the amplitude increased by 30%. Reducing the glottis diameter to 0.1 mm with 20% of tracheal elongation decreases the harmonic resonance frequency to approximately 3 kHz (figure 5), and vocal tract harmonic resonance can cover a wider range of frequencies where sparrows sing (1–8 kHz) [31].

Unfortunately, the exact changes in glottis form and size during vocalization remain largely unknown. Quantifying the motions of the glottis *in vivo* during vocalization would allow us to describe its motions in detail and check if it closes completely. If it closes totally, it will question the harmonic resonance assumption stating that the vocal tract is an open-end tube. In that case, harmonic resonance frequencies of the tube would drastically decrease when the glottis closes, resulting in a more significant variation of the harmonic resonance frequency, which could be an effective mechanism for sound modulation.

### 3.4. Tongue

In spite of what we could expect from previous work [9,19], we observed no specific changes in amplitude or frequencies when the tongue was added to the model. It might result from the tongue being simulated as a solid object, and thus can only shift in position but not vary in size.

The dynamical change of the tongue could affect frequency and amplitude modulation. If a distinct pattern of deformation was known, we could add it to our model, but to our knowledge, changes in the shape of the sparrow tongue are not documented yet. Future research should apply the cutting-edge methods recently used in mammals, such as X-rays, to measure the deformation of the tongue during bird vocalization, e.g. [35].

### 3.5. Oropharyngeal-oesophageal cavity

Our simulations not only confirmed the importance of the OEC volume variation in sound modulation [14,19] but also demonstrated that the OEC geometry must be considered when investigating sound filtration: both the length and the radius of a constant volume cylinder affect the resonance frequency and amplitude differently. We confirmed the previous numerical models [14,19] showing that the OEC volume variation can add at least one harmonic resonance to the vocal tract between 1 and 8 kHz: when OEC volume increases, the sound harmonic frequency decreases, which is in line with previous experimental studies and computational models [14,19]. Our results (figure 4*a*) showed three harmonic resonances for the OEC in volumes larger than 1.2 ml and two resonances for the lower volumes in the range of simulated frequencies (1 to 8 kHz). The first harmonic resonance is in the range of 1.70 to 2.90 kHz (figure 4*a*) and the second harmonic resonance is in the range of the tracheal harmonic resonance (arrow, figure 4*a*). This reveals that the OEC, together with the other structures of the vocal tract, can tune the primary produced sound throughout the whole frequency range in which a house sparrow typically sings (1–8 kHz) [31]. In addition, we found that the length variation tends to shift the harmonic resonances forward, with a constant amplitude, while the radius variation also decreases the first harmonic resonances amplitudes. We know that OEC can take a complex shape while the bird is singing [14,19]. Thus, we need a precise model of the anatomy of the OEC to find the exact contribution of the OEC to sound modulation.

When the trachea is modelled as a 36 mm tube [14], the tracheal harmonic resonance falls below the OEC's first harmonic resonance [14]. In our model, based on the realistic shape of the vocal system, the trachea is 30.5 mm long, which leads to a lower harmonic resonance frequency, falling in the range of the OEC second harmonic resonance. This illustrates the importance of incorporating accurate anatomical parameters in simulations, considering how a small variation in tracheal length may have significant impact on sound modulation. In a singing bird, both the trachea and the OEC vary in length and volume simultaneously, therefore the tracheal harmonic resonance could interact in the range of the first and second OEC harmonic resonance.

All these complex mechanisms produce the final shape and volume of the sparrow vocal system, which gives the sparrow ability to tune all the produced sound frequencies to their vocal system resonances. Our computational model of the sparrow's vocal system allowed us to test the hypothesis that the relative contributions of different elements of the avian upper vocal tract change across the spectral range, between 1 and 8 kHz. We showed that, by controlling the OEC volume, the sparrow could adjust the first harmonic resonance of OEC and tune over the low-range frequencies (1.5–3 kHz). The combination of the tracheal, glottis and beak movement can tune in the middle range (3–6 kHz), while the third harmonic resonance of the OEC tunes at higher frequencies (6–8 kHz) (figure 5). Thus, sparrows have the possibility and ability to synchronize all the produced sound frequencies to their vocal system resonances by controlling the vocal volume, especially the OEC volume, which is not directly in line with the upper vocal tract. This highlights the complexity of avian vocalization.

## 4. Material and methods

The dimensions of the trachea, larynx, beak, tongue and other anatomical features derive from CT scan images of a whole-body specimen of a house sparrow, preserved in 70% ethanol and stained with iodine-potassium iodine dissolved in water for four weeks (Morphosource: usnm:birds:657964 *P. domesticus*—ark:/87602/m4/M115379). The device used to acquire these images was a General Electric phoenix v|tome|x m, Smithsonian Institution Bio-Imaging Research (SIBIR) Center (National Museum of Natural History, Smithsonian Institution), with X, Y and Z pixel spacing of 0.067805 mm. The contrast agent used to prepare the specimen allows for the visualization of soft tissues (tongue, cheeks, etc.) and provides accurate modelling of the entire three-dimensional vocal system shape, in place. This specimen was used to investigate the chemical effects of staining [36], therefore it corresponds to a well-preserved specimen, prepared in optimal condition for CT-scan acquisition. Bone demineralization occurs throughout the staining process [36], but we chose a CT-scan performed after four weeks of staining, which minimizes the risk of tissue degradation while providing a satisfactory three-dimensional model quality. We segmented the CT scan, using Avizo (v. 9.7, FEI Visualisation Sciences Group, Burlington, MA, USA). We extracted the upper beak, lower beak, tongue and rest of the body, as separate three-dimensional objects. To save solver time without losing any accuracy, we reduced the three-dimensional object to the upper body only, and smoothed the external body, while preserving the entire trachea and the rest of the upper vocal tract.

### 4.1. Geometrical model

We imported the three-dimensional objects into COMSOL [37] Multiphysics software to build the initial model, which we digitally modified to test the influence of each structure on sound modulation (figure 1*a*). The initial model has a 30.58 mm long trachea and a 10° beak gape with the larynx opening, the glottis, approximately 0.7 mm in radius.

#### 4.1.1. Trachea

To assess the effect of trachea shortening, we cut 7%, 11% and 20% of the initial model trachea (figure 1*b*). We modelled extreme elongation or shortening to test the potential effect of trachea lengthening; our personal observations on cadavers led us to test a maximum of 20% variation. We used the ‘differences’ tool in COMSOL Multiphysics software [37]. We considered the end correction and added a short distance to the actual length of the beak [38,39].

#### 4.1.2. Glottis

We started with a 0.5 mm glottis diameter, corresponding to the geometry of the µCT scan. For that, we inserted a cylinder in the original larynx and cut the cylinder surface area to decrease the diameter of the glottis. It resulted in a sharp edge at the junction of the glottis and the cylinder. We compared the result for a geometry with a smooth surface or a sharp edge. The difference was inferior to 1%. Therefore, we considered that the effect was negligible. With this method, we decreased the radius by 0.1 mm steps to reach a final radius of 0.1 mm (figure 1*c*).

#### 4.1.3. Tongue

As the tongue was previously segmented, we were able to move it in COMSOL Multiphysics software, using the rotation tool [37]. We considered the tongue in three different positions: (i) the tongue touches the lower beak, (ii) the tongue is located in the middle of the beaks, and (iii) the tongue touches the upper beak. We chose these conformations to mimic the potential motions of the tongue during vocalization, from the lower to the upper beak. We compared those three models with the initial model without tongue (figure 1*d*).

#### 4.1.4. Beak gape

We imported the beak three-dimensional models derived from the segmentation, in Autodesk Maya (Student v. 2020). We placed a virtual joint at the approximate location of the lower beak joint [24] (figure 1*e*). We chose three beak poses, representing extreme beak gape angles: 10°, 20° and 30°.

#### 4.1.5. Oropharyngeal-oesophageal cavity

**Figure 6.**
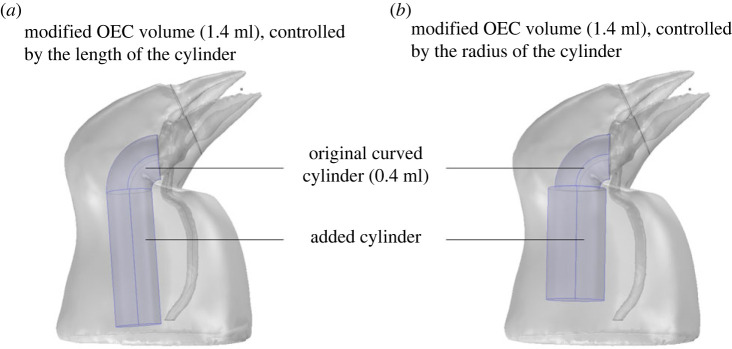
Tested OEC models. The total volume of OEC (1.4 ml) is controlled by the length (*a*) or the radius (*b*) of a cylinder, added to an original curved cylinder of 0.4 ml.

Based on the location and shape of the oropharyngeal cavity (OEC) on the µCT-scan data, we artificially added a curved cylindric shape of 0.4 ml to the initial model (figure 6). To investigate the role of the three-dimensional shape on sound modulation, we modelled the OEC in seven different volumes (1.4, 1.3, 1.2, 1.1, 1, 0.9 and 0.8 ml) (figure 4), by adding a cylindrical shape to the initial curved cylinder and by incrementally changing its length: 8, 10, 12, 14, 16, 18 and 20 mm (figure 6*a*), and radius: 2.5, 2.8, 3.1, 3.34, 3.57, 3.78, 4 mm (figure 6*b*). We estimated the volume of the OEC using previous X-ray video recordings of OEC movements during vocalization [14,15,19]. We checked the effect of the connection between the cylinder and torus, and between the torus and the buccal cavity in each model. The effect was inferior to 1%, thus considered ignorable.

### 4.2. Computational model

We used the COMSOL Multiphysics [37] software to create our model and perform the modelling, available in electronic supplementary material, S1. We used pressure acoustics in the frequency domain interface. This domain is designed for the analysis of various types of pressure acoustics problems in the frequency domain, all concerning pressure waves in a fluid. The frequency-domain formulation uses an inhomogeneous Helmholtz equation (COMSOL Multiphysics v. 5.6. Reference Manual, 2018),

4.1 $$\nabla \cdot \left(-\frac{1}{\rho_{c}}(\nabla p-q_{d})\right)-\frac{\omega^{2}p}{\rho_{c}c_{c}^{2}}=Q_{m},$$

where ‘*ρ*’ (kg m^−^^3^) is density and ‘*p*’ (Pa) is pressure, ‘*c*’ (m s^−1^) is the speed of sound in the medium, ‘*ω*’ (rad s^−1^) is the angular frequency and *q* (N m^−^^3^) and Q (s^−^^2^) are dipole and monopole sources, respectively. The subscript ‘c’ in ‘*ρ*_c_’ and ‘c_c_’ denotes that these can be complex-valued quantities, and *ω* = 2*πf*, where *f* (Hz) denotes the frequency. With this formulation, we can compute the frequency response of a system for chosen frequencies.

To complete computation, boundary conditions have to be defined. Several boundary conditions are used for the sparrow model, such as sound hard boundary and impedance condition for airways, the radiation boundary condition for the edge of the model, and pressure for sound input. Compressible lossless (no thermal conduction and no viscosity) conditions are assumed.

#### 4.2.1. Sound hard boundaries

A sound-hard boundary is a boundary where the normal component of the acceleration is zero (COMSOL Multiphysics v. 5.6. Reference Manual, 2018),

4.2 $$-n\cdot \left(-\frac{1}{\rho_{0}}(\nabla p-q)\right)=0.$$

In the sparrow model, zero dipole charge and constant fluid density are assumed, so the normal derivative of the pressure will be zero at the boundary,

4.3 $$\frac{\partial p}{\partial n}=0.$$

#### 4.2.2. Impedance boundary

In the impedance boundary, part of the wave is reflected from the boundary, and the other part is transmitted across the boundary. This boundary condition is a generalization of the sound hard and soft boundary conditions (COMSOL Multiphysics v. 5.6. Reference Manual, 2018),

4.4 $$n\cdot \left(-\frac{1}{\rho_{0}}(\nabla p-q)\right)-\frac{i\omega p}{Z}=0,$$

where *Z* (Pa s m^−1^) is the acoustic input impedance of the external domain.

#### 4.2.3. Radiation boundary condition

To avoid the effect of reflection of outgoing waves the radiation boundary is assumed for the outer layer of the surrounding area. This boundary condition allows an outgoing wave to leave the modelling domain with minimal reflections. The radiation boundary conditions read (COMSOL Multiphysics v. 5.6. Reference Manual, 2018)

$$\begin{array}{rl} & -n\cdot \left(-\frac{1}{\rho_{c}}(\nabla p_{t}-q_{d})\right)+\left(ik_{\mathrm{eq}}+\frac{1}{r}\right)\frac{\;p}{\rho_{c}}-\frac{r\Delta ∥p}{2\rho_{c}(ik_{\mathrm{eq}}r+1)}\\ & =-\frac{r\Delta ∥p_{i}}{2\rho_{0c}(ik_{\mathrm{eq}}r+1)}+\left(ik_{\mathrm{eq}}+\frac{1}{r}\right)\frac{\;p_{i}}{\rho_{c}}+n\cdot \frac{1}{\rho_{c}}\nabla p_{i}.(4.5) \end{array}$$

In the spherical wave cases, ‘*r*’ is the shortest distance from the point *r* = (*x*, *y*, *z*) on the boundary to the source. Δ∥ at a given point on the boundary denotes the Laplace operator in the tangent plane at that particular point. The boundary condition is based on an expansion in spherical coordinates from Bayliss *et al*. [40], implemented in the second order.

#### 4.2.4. Pressure source

Syrinx sound output is considered model input, which is modelled by the pressure source. With this boundary condition, constant acoustic pressure is maintained at the boundary,

$$p=p_{0}.$$

### 4.3. Sound pressure level

To be able to compare the results of the simulations, we used the sound pressure level (SPL) definition (COMSOL Multiphysics v. 5.6. Reference Manual, 2018). SPL is the pressure level of a sound. It is measured in dB. The zero level on the dB scale depends on the type of fluid, which is called reference pressure. SPL is equal to

4.6 $$L_{p}=20\log\left(\frac{\;p_{\mathrm{rms}}}{\;p_{\mathrm{ref}}}\right)\;\mathrm{with}\;p_{\mathrm{rms}}=\sqrt{\frac{1}{2}pp^{*}},$$

where *L*_p_ is sound pressure level, *p*_ref_ is the reference pressure, and the star (*) represents the complex conjugate. In the sparrow model, the reference sound pressure is for air and equal to 20 µPa and always *L*_p_ is measured at the fixed distance in the middle of the beaks.

### 4.4. Mesh

The wavelength is dependent on the frequency and the speed of sound. In the current model, frequencies ranging from 1 to 8 kHz are simulated. The lowest wavelength occurs for the highest frequency of 8 kHz. The maximum mesh size should not be lower than five times the wavelength [41,42]. Based on the *h*_max_ = *c*_0_/*f*_max_/5 our maximum mesh element size should be 0.0086 m long. The model of a sparrow with 10° of beak gape consists of 20 105 domain mesh elements, 3250 boundary mesh elements and 161 mesh edge elements.

### 4.5. Parameters effects and set-up

We computed vocal tract harmonic resonance from 1 to 8 kHz of frequencies where sparrows sing [31]. To be able to compare the results, we calculated the total sound pressure level on the scale of dB in the fixed position at an equal distance from the end of the upper beak (figure 1).

#### 4.5.1. Pressure input

The input pressure is set on 1 Pa, which is equal to 94 dB for all the frequencies in the entrance of the trachea for all the models.

#### 4.5.2. Trachea boundary conditions and materials

**Figure 7.**
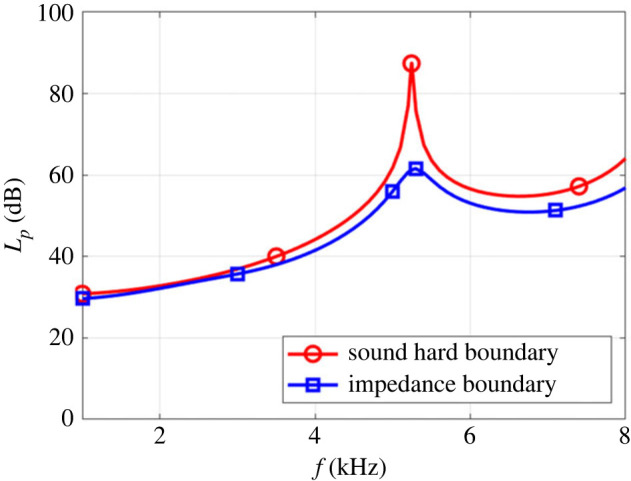
Total sound pressure level in the scale of dB versus frequency from 1 to 8 kHz in two boundary conditions.

The trachea is composed of rings of cartilage and muscles, which are not considered as hard boundaries. We calculated the total sound pressure but considered the trachea as soft tissue, like muscles, and applied an impedance boundary condition (figure 7).

As displayed, the total pattern of frequency at the end of the beak is the same for both boundary conditions, but the sound hard boundary has a higher amplitude compared with soft boundary conditions. For the rest of our work, an impedance boundary condition for the airways system with skin materials properties is considered (except for beak that has bone properties). Reflection in the hard boundary condition is different from the impedance boundary condition. Waves have momentum and energy, and whenever a wave encounters an obstacle, it is reflected by the obstacle. When a sound wave encounters a rigid wall, it bounces back, and this ‘reflection’ of the object can be analysed in terms of momentum and energy conservation. If we consider the collision between wave and wall perfectly elastic, then all the incident energy and momentum are reflected. In this case, the wave bounces back at the same speed. If the collision is inelastic, like what we have in the trachea with muscle and cartilage, then the wall of the trachea (or wave) absorbs some of the incident energy and momentum and the wave does not bounce back with the same speed.

#### 4.5.3. Trachea length

Trachea length has a reverse relationship with the harmonic resonance of the tube. As in the equation below, which is for ideal cylindrical tubes shows, decreasing tube length increases the harmonic resonance frequency (*f*).

4.7 $$f=\frac{c}{2L+\Delta L},$$

where *L* is tube length and Δ*L* is end correction. At low frequencies, the velocity potential inside the tube is the same as if the tube is extended by a certain fraction of the radius and the open end behaves like a loop. The exact value of the end correction turns out to be 0.6133 × *r*, where *r* is the hydraulic radius [39]. Recent hypotheses consider the songbird vocal tract as a rigid tube with a specific harmonic resonance [21]. If we consider the bird's trachea as a perfect tube with the same length and average diameter equal to the initial trachea model, the theory shows a 2 mm shortening of length (7%), shifting forward 0.39 kHz of the harmonic resonance frequency [39]. In other words, in theory, 7% shortening increased 7% of the harmonic resonance frequency, while if we consider the form of all of the vocal system, shortening 7% of the length of the trachea increases 2% of the harmonic resonance frequency of the upper vocal tract, as found by the computational model.

During each simulation, all the organs are assumed to be solid and not change their forms unless it is mentioned.

## Data Availability

All data used for the analyses are available from the Dryad Digital Repository: https://doi.org/10.5061/dryad.msbcc2g1k [43].
